# The Perceptions of Male Accessibility to the Fields of Nursing Practice by Those Studying or Teaching Nursing in England: Cross‐Sectional Survey

**DOI:** 10.1111/jan.16789

**Published:** 2025-02-03

**Authors:** Daniel Carter, Lucian Hadrian Milasan, Andrew Clifton, George Mcgill, Julian Stribling, Kay De Vries

**Affiliations:** ^1^ Faculty of Health and Life Sciences De Montfort University Leicester UK; ^2^ Institute of Health and Allied Professions Nottingham Trent University Nottingham UK; ^3^ School of Health and Sports Science University of Suffolk Ipswich UK; ^4^ Statistical Contractor Rsquaredlabs Littlehampton UK; ^5^ Nightingale Faculty of Nursing, Midwifery and Palliative Care Kings College London London UK

**Keywords:** gender, male access, men in nursing, nursing, nursing education

## Abstract

**Aims:**

Investigate the perception of male accessibility to the fields of nursing practice by those studying or teaching nursing in England.

**Design:**

Cross‐sectional survey.

**Methods:**

Online questionnaire with three closed‐scale questions and two open‐text questions designed to elicit perceptions on the accessibility of men to the fields of nursing practice. The questionnaire was distributed to the staff and students at 61 nursing schools in England. Inferential and descriptive statistics were used to analyse the closed questions data and inductive content analysis was used to analyse open‐text questions data.

**Results:**

Students (*n* = 52) and staff (*n* = 51) responded to the survey. Adult (Mdn = 6, IQR = 2) and mental health (Mdn = 6, IQR = 2) were perceived as the most accessible fields of nursing practice to men, and child (Mdn = 4, IQR = 2) the least. Specialised practice areas in acute and emergency (Mdn = 6, IQR = 2), education (Mdn = 6, IQR = 2), leadership (Mdn = 7, IQR = 1), prison services (Mdn = 7, IQR = 1), and research (Mdn = 7, IQR = 2) were rated the most accessible to men and neonatal care (Mdn = 3, IQR = 3) the least. Societal stereotyping and stigma were seen as barriers to men entering the nursing profession. The perception that nursing is a feminised profession persists and a distrust of men is associated with child nursing. Men were viewed as progressing to leadership roles with greater ease than women.

**Conclusion:**

Societal level stereotyping and stigma are perceived as prevalent in nursing practice areas considered less accessible to men entering the nursing profession.

**Impact:**

This study adds insight into the gendered nature of nursing and highlights the barriers to men entering a profession with a workforce crisis.

**Reporting Methods:**

STROBE cross‐sectional studies guidelines. COREQ guidelines for content analysis.

**Patient or Public Contribution:**

No patient or public contribution.

## 1 Introduction

The nursing sector is one of the most gender‐segregated sectors in the United Kingdom (Clayton‐Hathway et al. 2020) with men accounting for just 11% of the 771,445 registered nurses (Nursing and Midwifery Council 2022). Student nursing programmes in UK Higher Education Institutes (HEI) also comprise a large gender imbalance with men accounting for only 12% of applicants accepted onto nursing programmes, and as such “*men remain an untapped source*” of the future workforce (Universities and Colleges Admissions Service [UCAS] 2023 and Health Education England [HEE] 2021, 5). Globally, there is an ongoing call to develop an understanding of why low numbers of men choosing a career in nursing persists and to create strategies that encourage more men into the profession (Whitford et al. 2020; Punshon et al. 2019; Clayton‐Hathway et al. 2020; Office for Students 2020).

Increasing the uptake of the nursing profession by men is an important aim that can bring benefits to the sector. First, increasing male uptake will help deal with the global chronic shortage of nurses (Clayton‐Hathway et al. 2020; UCAS/HEE 2021), that persists in the UK National Health Service (NHS) exacerbated by the Covid‐19 context: as of March 2023, over 43,000 nursing vacancies have been recorded in the NHS in England (NHS England 2023). Second, an increase of men working as nurses diversifies perspectives, behaviours, and values of a healthcare workforce (Brody et al. 2017) which has the potential to improve the quality of care delivered due to the broadening of knowledge, skills, perspectives, and experiences offered by the workforce (Shea‐Lewis 2002). Heeding the calls to develop a culture that brings diverse skills, perspectives, and communication styles to student nursing programmes can translate into a future workforce where the benefits of diversity are experienced at the level of patient care (Bleich, MacWilliams, and Schmidt 2015). Third, increasing men in nursing can be a means by which to improve the working conditions for the nursing workforce as a whole as there tends to be a perception (underscored with reality) that jobs where the majority of the workforce are women tend to be lower valued and thus less well remunerated (Waters 2002). Finally, given that men account for about 50% of the population, attracting more men into the profession will be a step towards reflecting current gender demographics in society and reduce the burden of formal and informal care from women (Clifton, Crooks, and Higman 2020).

Strategies for increasing the uptake of the nursing profession by men have been proposed. For example, in New Zealand second‐degree graduate entry programmes could prove more attractive to the profession for mature men, who had undertaken other routes into nursing beforehand (Harding et al. 2018). Raising awareness of the diversity of roles on offer in the nursing profession has also been proposed, such as specialised technical roles, such as in Intensive Therapy Unit (ITU) and Emergency Department (ED) (Whitford et al. 2020; Guy, Hughes, and Ferris‐Day 2022). Elsewhere, importance is placed on addressing the long‐standing stereotyping and stigma associated with men in care‐based professions as a means of attracting men more broadly to these sectors (Teresa‐Morales et al. 2022; Loughrey 2008). Such strategies are crucial in re‐shaping nursing into a more inclusive and diverse profession with regard to men working within the profession.

## 2 Background

Men have a long history of contributing to nursing since the fourth and fifth centuries when men in monastic communities cared for the sick and dying (Evans 2004). In the eighteenth century, men also provided nursing care to male patients in charity hospitals, asylums, and workhouses (Mackintosh 1997). However, by the nineteenth century, nursing in the United Kingdom developed a more feminine identity, a shift exemplified in 1848 when Fraser's magazine described ideal nurses as “women of patience, gentleness and self‐devotion” (Williams 1974, as cited by Mackintosh 1997, 233). Florence Nightingale, a key figure in modern nursing, strongly believed that nursing required “feminine” traits that she deemed essential (Clifton, Crooks, and Higman 2020; Evans 2004; Mackintosh 1997; Brown, Nolan, and Crawford 2000). With the Nurse Registration Act in 1919, only women could fully register as nurses, reinforcing nursing as a female profession during a time when women's occupational choices were limited (Flaskerud and Halloran 2018). However, men continued working in psychiatric institutions, often perceived as warders rather than nurses, which may explain the higher male presence in the contemporary mental health nursing (Brown, Nolan, and Crawford 2000). Some authors argue that nursing's feminine identity arose within the wider movement of feminism (Hoffmann 1991) as a counterbalance to the masculine battlefield space of the World Wars (Brooks 2019), while others interpret this as an expression of gender liberation from male‐doctor dominance and female subservience (Garcia and Qureshi 2022; Clifton, Crooks, and Higman 2020).

The perception of nursing as “women's work” has reinforced gender stereotypes that discourage men from entering the field (Ross 2017). Stereotyping impacts both young men and mature students (Whitford et al. 2020), with the media further deepening this divide by focusing on female objectification and the lack of male role models in nursing (Weaver et al. 2014). Men entering the nursing workforce navigate a sector with complex gender dynamics where they face both challenges and successes of being a visible minority (Smith et al. 2021). Male nurses are often stereotyped as homosexual and associated with traits such as warmth, compassion, and nurturing (Loughrey 2008; Teresa‐Morales et al. 2022), although some men accept these associations to attain higher management roles (Liu and Li 2017).

Gender bias also leads to distrust towards men in nursing, with male nurses perceived as a potential threat, leading to patient discomfort and avoidance (Harding, North, and Perkins 2008; Teresa‐Morales et al. 2022). Men experience isolation in nursing education, contributing to lower completion rates due to an uncomfortable environment during academic and clinical training when compared to women (Stott 2007; Mclaughlin, Muldoon, and Moutray 2010). Guy, Hughes, and Ferris‐Day (2022) found that male students often feel isolated due to nursing's socially constructed feminine image, which conflicts with traditional masculinity. This can lead to “soft masculinity,” where men in nursing find their feminine traits emphasised, creating cognitive dissonance with societal gender expectations (Holyoake 2002). A study by Ramjen et al. (2024) showed misconceptions of men in nursing to be very low amongst Australian nursing students, although participants who were more likely to hold generalised views of men in the nursing profession were male students.

Financial security is often a motivation for men entering nursing, but the perception of the profession as being a low status deters others (Ashkenazi et al. 2017). Men in the NHS are also more likely to occupy senior roles and higher pay grades, despite the predominance of women in nursing (Punshon et al. 2019). Certain nursing specialties like mental health and emergency roles are seen as suitable for men due to associations with physical strength or technical skills (Evans 1997; Hurley et al. 2022). While fields such as mental health nursing initially attracted more men, recent statistics suggest a decline of 29% in male representation potentially as a consequence of the expansion of community psychiatric services (NHS 2014).

While some studies have explored male access to specific nursing pathways, none have examined perspectives from students and lecturers in English HEI. Understanding how accessibility varies for men could help develop strategies to promote diverse nursing career paths, potentially increasing male participation in the profession.

## 3 Study Aims

This study aimed to examine the accessibility of men to the fields of nursing practice (based on the Nursing and Midwifery Council criteria for fields of practice), and specialised areas of nursing, as perceived by a group of those currently studying or teaching nursing in England. The fields of nursing practice examined are adult, child, learning disability, and mental health. The specialised areas examined are leadership and management, acute and emergency care, intensive and critical care, acute wards, community and primary care, public health, prison services, neonatal, clinical commissioning, education and training, research and development, residential care, and palliative care. The specific questions this study aims to address are as follows:

- How are men perceived in terms of their accessibility to practising in the various fields of nursing practice?
- How are men perceived in terms of their suitability to practising in the various fields of nursing practice?
- How are men perceived in terms of their accessibility to practising in the various specialised areas of nursing?

By accessibility, we mean being able to reach and enter the various fields of nursing as a nursing practitioner. By Suitability, we mean the various fields of nursing being considered acceptable and appropriate to enter as a nursing practitioner.

This study has been approved by the ethics committee of the Faculty of Health and Life Sciences at De Montfort University (485562).

## 4 Methods

### 4.1 Design

A cross‐sectional survey was used to undertake this study. An online questionnaire was administered using a questionnaire, which included closed and open questions that allowed for an unlimited text response.

The questionnaire was designed by the researchers with questionnaire items deriving from the literature review and from the study questions. Four of the five researchers involved in the development of the questionnaire have a background in nursing in practice, teaching and research. The questionnaire was piloted with seven academics (female = 3, male = 4) with a background in a variety of nursing clinical practice fields, and nursing teaching and research. The academics piloting the questionnaire provided feedback on questionnaire items that included improving the clarity of questions that were said to have ambiguity, particularly around the terminology used. In response to feedback the questionnaire was amended, for example, clarity regarding nursing roles was improved.

Three Likert‐scale type closed questions were designed to quantify perceptions of how accessible the various fields of nursing practice and specialised areas of the nursing profession are to men. The first closed question comprised four statements, the second closed question comprised 13 statements, and the third closed question comprised four statements. For the first closed question respondents were asked to indicate, on a scale of 1–7, with 1 indicating “Not at all accessible” and 7 indicating “Extremely accessible,” as a profession, how accessible to men each of the fields of nursing practice are. For the second closed question, respondents were then asked to indicate how accessible individual specialised areas of nursing practice are to men, on a scale of 1–7, with 1 being “Not at all accessible” and 7 being “Extremely accessible.” For the third closed question, respondents were then asked to indicate how well suited they think men are to practice in each of the fields of nursing practice, on a scale of 1–7, with 1 being “Not at all suited” and 7 being “Very well suited.”

To gather more in‐depth data on respondents' views, two unlimited open‐text questions were designed to allow for nuances of perceptions and experiences of participants to be captured in the data. Respondents were asked to share in unlimited open text response boxes any reasons why they think any of the nursing fields of practice, or any of the specialised areas may or may not be accessible to men to enter as a profession. Seven further questions collected respondent demographic information.

### 4.2 Data Collection

A list of schools in HEIs across England where nursing is taught was compiled, and the questionnaire was distributed by email to the school head (*n* = 61). The online questionnaire was open to respondents from 22 February 2023 to 22 March 2023. Department heads were invited to promote the questionnaire with their respective staff and student populations from all nursing fields. All study respondents self‐selected, and the respondent invitation clarified that any nursing staff or student interested could participate. Respondents' informed consent was obtained by asking them to confirm they had read and understood the survey participation sheet and agreed to participate.

### 4.3 Data Analysis

Descriptive and inferential statistical techniques were applied to analyse the quantitative scale data. Comparative techniques included: the Friedman Test for testing any observed difference of respondents' perception between the fields of nursing practice; the Wilcoxon signed‐rank test to conduct post hoc analysis to determine which specific pairs of variables difference is observed between; the Mann–Whitney *U* test to identify any differences between academic staff and student respondents, and female and male respondents.

Inductive content analysis was used to analyse data from open text questions. An inductive approach enabled general statements and themes to be developed from specific data extracts (Elo and Kyngäs 2008). One of the decisions in content analysis is determining the unit of analysis that conveys the focus of the study (Elo and Kyngäs 2008). For this study, the unit of analysis was either a word, a phrase, a sentence, or a paragraph of text from the open text responses and the unit of analysis was analysed as manifest content. The process, completed by the first author, was an initial comprehensive reading of the text to build understanding and generate initial ideas of what respondents were expressing, followed by organising, condensing, coding, and categorising the data, a method that takes the respondents' responses through levels of abstraction (Erlingsson and Brysiewicz 2017). To ensure reliability, the last author then undertook a comprehensive reading of the text and confirmed the coding applied by the first author. The first and last authors together then generated themes based on the grouping of codes.

## 5 Results

### 5.1 Respondent Characteristics

The sample size for this study was 106. This sample was taken from the population of all nursing staff and nursing students across nursing schools based in England. Table 1 displays the respondent characteristics. The majority of respondents were female (61%) and male representation was (39%). The gender profile of academic staff teaching nursing in England could not be found and determining the gender profile across all nursing programmes was also not possible due to lack of available data in this area. The proportion of respondents who were nursing students and nursing academic staff was close to even. All respondents were either teaching or studying at a HEI based in England. Determining the total nursing student population in England across all years and programmes of study for both undergraduate and postgraduate has not been possible. It has also not been possible to determine the population size for nursing academic staff across nursing schools in England. Calculating a response rate for the population of interest for this study has therefore not been possible.

**TABLE 1:** Questionnaire respondent characteristics.

Characteristic	*n*	%
Student or academic		
Student	52	50.5
Academic	51	49.5
Gender		
Female	63	60.6
Male	40	38.5
Non‐binary	1	1.0
Age group		
18–24	14	13.7
25–34	29	28.4
35–44	21	20.6
45–54	14	13.7
55–64	24	23.5
Ethnicity		
White (British)	83	83.8
Ethnicity minority groups	16	16.2
Region of study/work		
London	20	19.0
East Midlands	25	23.8
East of England	8	7.6
North East	19	18.1
North West	3	2.9
South East	9	8.6
South West	8	7.6
West Midlands	13	12.4

Abbreviations: *n*, total number of observations per respondent characteristic; %, proportion of observations per respondent characteristic.

### 5.2 The Perception of the Accessibility of Men to the Fields of Nursing Practice

Based on median scores respondents rated Adult Nursing (Mdn = 6, IQR = 2) and Mental Health Nursing (Mdn = 6, IQR = 2) as the most accessible fields, and Child Nursing the least accessible field (Mdn = 4, IQR = 2) (Table 2).

**TABLE 2:** Respondent median scores rating male accessibility to each field of nursing.

Nursing field	All respondents			Student			Academic			Female			Male		
	*N*	Mdn	IQR	*n*	Mdn	IQR	*n*	Mdn	IQR	*n*	Mdn	IQR	*n*	Mdn	IQR
Adult	106	6.0	2	52	6.0	1	51	6.0	2	63	6.0	2	40	5.5	3
Child	106	4.0	2	48	4.0	2	50	3.0	3	59	4.0	3	39	3.0	2
Learning disability	100	5.5	3	50	5.0	2	47	6.0	3	58	6.0	3	39	5.0	2
Mental health	103	6.0	2	50	6.0	3	50	7.0	1	60	6.0	2	40	6.5	2

Abbreviations: IQR, interquartile range; Mdn, median score; *N*, total number of respondents; *n*, total number of respondents per comparison group.

In order to compare if the observed difference in the respondents' perception of male accessibility to the different fields of nursing was of statistical significance, a Friedman test was applied to the data. Results showed a statistically significant difference in the mean rank of scores across the fields of nursing groups *χ* ^2^(3, *n* = 96) = 130.08, *p* < 0.001, revealing the difference observed between the variables to be statistically significant. To determine between which fields of nursing the statistically significant difference is, and the effect size of any difference, post hoc analysis was conducted using a Wilcoxon signed‐rank test using a Bonferroni adjusted alpha level (*p* = 0.008). The test revealed that respondents perceive Child Nursing to be less accessible to men than Adult Nursing, a result that is statistically significant (*p* < 0.001), with a large effect size (*r* = 0.51) (Table 3). The test also revealed that respondents perceived Child Nursing to be less accessible to men than Mental Health Nursing, a result that is statistically significant (*p* < 0.001), with a large effect size (*r* = 0.51). While other fields had accessibility rating differences that were statically significant, effect sizes were medium or small (*r* < 0.50). The strength of the effect size was interpreted based on Cohen's (1988) criteria.

**TABLE 3:** Wilcoxon signed‐rank test results comparing the difference in respondents perception rating of male accessibility between each field of nursing practice.

Nursing field 1	Nursing field 2		*N*	Mean rank	*z*	*p*	*r*
Child	Adult	Negative ranks	69^a^	35.84	−7.26	< 0.001	0.51
		Positive ranks	1^b^	12.00			
		Ties	31^c^				
Child	Mental health	Negative ranks	72^a^	37.76	−7.22	< 0.001	0.51
		Positive ranks	2^b^	28.00			
		Ties	25^c^				
Learning disability	Adult	Negative ranks	32^a^	24.47	−2.37	0.02	0.17
		Positive ranks	15^b^	23.00			
		Ties	53^c^				
Learning disability	Mental health	Negative ranks	37^a^	19.31	−5.11	< 0.001	0.36
		Positive ranks	1^b^	26.50			
		Ties	60^c^				
Child	Learning disability	Negative ranks	58^a^	34.05	−6.35	< 0.001	0.45
		Positive ranks	6^b^	17.50			
		Ties	34^c^				
Mental health	Adult	Negative ranks	13^a^	21.00	−2.65	0.01	0.19
		Positive ranks	31^b^	23.13			
		Ties	59^c^				

*Note:* Results of testing the null hypothesis that there is no difference in the perception of male accessibility between the various fields of nursing practice.

Abbreviations: *z* = standardised test statistic, *r* = effect size according to Cohen (1988), the *p*‐value (*p*) significance level is set at equal to or less than 0.05.

^a^ The number of cases where the scores were lower for nursing field 1 than nursing field 2.

^b^ The number of cases where the scores were higher for nursing field 1 than nursing field 2.

^c^ The number of cases where there was no difference between nursing field 1 and nursing field 2.

### 5.3 Comparison by Staff/Student and Gender

A Mann–Whitney *U* test was applied to the data to determine whether any difference between the median scores of academic staff and student respondents were of statistical significance regarding the accessibility of men to the fields of nursing. The test revealed the fields of Mental Health Nursing to be perceived as less accessible to men by students (Mdn = 6, IQR = 3, *n* = 47) compared to academic staff (Mdn = 7, IQR = 1, *n* = 50), *U* = 826, *z* = −3.09, *p* = < 0.01, *r* = 0.31, with a medium effect size (Cohen 1988) (Table 4). While the test also revealed the field of learning disability nursing to be perceived as less accessible to men by students (Mdn = 5, IQR = 2, *n* = 47) compared to academic staff (Mdn = 6, IQR = 3, *n* = 50), *U* = 879, *z* = −2.20, *p* = 0.03, *r* = 0.22, the effect size was small (Cohen 1988).

**TABLE 4:** Mann–Whitney *U* test results comparing the perception rating of male accessibility to each field of nursing practice between the academic nursing staff and nursing student respondents.

Nursing field	Respondent group	*n*	Mean rank	*U*	*z*	*p*	*r*
Adult	Academic	52	57.46	1047.5	−1.90	0.06	0.19
	Student	51	46.64				
	Total	103					
Child	Academic	48	47.41	1095.5	−0.75	0.45	0.08
	Student	50	51.68				
	Total	98					
Learning disability	Academic	50	55.30	879	−2.20	0.03	0.22
	Student	47	43.08				
	Total	97					
Mental health	Academic	50	58.98	826	−3.09	< 0.01	0.31
	Student	50	42.02				
	Total	100					

*Note:* Results of testing the null hypothesis that there is no difference in the perception of male accessibility to the various fields of nursing practice between the student and academic respondent groups.

Abbreviations: *U* = primary test statistic, *z* = standardised test statistic, *r* = effect size according to Cohen (1988), the *p*‐value (*p*) significance level is set at equal to or less than 0.05.

A Mann–Whitney *U* test was also applied to the data to determine whether any difference between the scores of female and male respondents were of statistical significance regarding the perception of accessibility for men to the fields of nursing. While the test revealed that male respondents (Mdn = 3, IQR = 2, *n* = 39) perceived the field of Child Nursing to be less accessible to men compared to female respondents (Mdn = 4, IQR = 3, *n* = 59), *U* = 811, *z* = −2.50, *p* = 0.01, *r* = 0.25 (Table 5), the effect size was small (Cohen 1988).

**TABLE 5:** Mann–Whitney *U* test results comparing the perception rating of male accessibility to each field of nursing practice between the female and male respondents.

Nursing field	Respondent group	*N*	Mean rank	*u*	*z*	*p*	*r*
Adult	Female	63	53.98	1135.5	−0.87	0.38	0.09
	Male	40	48.89				
	Total	103					
Child	Female	59	55.25	811	−2.50	0.01	0.25
	Male	39	40.79				
	Total	98					
Learning disability	Female	60	53.01	898.5	−1.76	0.08	0.18
	Male	40	43.04				
	Total	100					
Mental health	Female	58	48.67	1090	−0.82	0.41	0.08
	Male	39	53.25				
	Total	97					

*Note:* Results of testing the null hypothesis that there is no difference in the perception of male accessibility to the various fields of nursing practice between the female and male respondent groups.

Abbreviations: *U* = primary test statistic, *z* = standardised test statistic, *r* = effect size according to Cohen (1988), the *p*‐value (*p*) significance level is set at equal to or less than 0.05.

### 5.4 The Perception of the Suitability of Men to the Fields of Nursing Practice

While the median scores of the respondents rating did not differ for how suitable each nursing field is to men (Table 6), results from a Friedman Test indicated a statistically significant difference in the mean ranks of scores across the fields of nursing groups *χ* ^2^(3, *n* = 102) = 36.84, *p* < 0.001. To determine between which fields of nursing the statistically significant difference is, and the effect size of any difference, post hoc analysis was conducted using a Wilcoxon signed‐rank test using a Bonferroni adjusted alpha level (*p* = 0.008). The results revealed a statistically significant difference between the fields of: Child Nursing and Adult Nursing (*z* = −4.29, *p* < 0.001) with a medium effect size (*r* = 0.30) (Cohen 1988); Child Nursing and Mental Health Nursing (*z* = −3.94, *p* < 0.001), although here the effect size was small (*r* = 0.27); Child Nursing and learning disability nursing (*z* = −2.92, *p* < 0.001), although here the effect size was small (*r* = 0.20) (Table 7).

**TABLE 6:** Respondents median scores rating male suitability to each general field of nursing.

Nursing field	All respondents			Student			Academic			Female			Male		
	*N*	Mdn	IQR	*n*	Mdn	IQR	*n*	Mdn	IQR	*n*	Mdn	IQR	*n*	Mdn	IQR
Adult	105	7.0	1	51	7.0	1	51	7.0	0	63	7.0	1	39	7.0	1
Child	103	7.0	2	50	7.0	2	50	7.0	1	61	7.0	2	39	7.0	2
Learning disability	103	7.0	1	50	7.0	1	50	7.0	0	62	7.0	1	38	7.0	1
Mental health	105	7.0	0	51	7.0	1	51	7.0	0	63	7.0	0	39	7.0	1

Abbreviations: *N*, total number of respondents; *n*, total number of respondents per comparison group; Mdn, median score; IQR, interquartile range.

**TABLE 7:** Wilcoxon signed‐rank test results comparing the difference in respondents perception rating of male suitability between each field of nursing practice.

Nursing field 1	Nursing field 2		*N*	Mean rank	*z*	*p*	*r*
Child	Adult	Negative ranks	24^a^	13.33	−4.29	< 0.001	0.30
		Positive ranks	1^b^	5.00			
		Ties	78^c^				
Mental health	Adult	Negative ranks	5^a^	8.40	−0.24	0.81	0.02
		Positive ranks	7^b^	5.14			
		Ties	93^c^				
Learning disability	Adult	Negative ranks	10^a^	6.75	−2.26	0.02	0.16
		Positive ranks	2^b^	5.25			
		Ties	91^c^				
Mental health	Child	Negative ranks	2^a^	8.75	−3.94	< 0.001	0.27
		Positive ranks	23^b^	13.37			
		Ties	78^c^				
Learning disability	Child	Negative ranks	4^a^	7.00	−2.92	0.004	0.20
		Positive ranks	16^b^	11.38			
		Ties	82^c^				
Learning disability	Mental health	Negative ranks	12^a^	6.75	−2.55	0.01	0.18
		Positive ranks	1^b^	10.00			
		Ties	90^c^				

*Note:* Results of testing the null hypothesis that there is no difference in the perception of male suitability between the various fields of nursing practice.

Abbreviations: *z* = standardised test statistic, *r* = effect size according to Cohen (1988), the *p*‐value (*p*) significance level is set at equal to or less than 0.05.

^a^ The number of cases where the scores were lower for nursing field 1 than nursing field 2.

^b^ The number of cases where the scores were higher for nursing field 1 than nursing field 2.

^c^ The number of cases where there was no difference between nursing field 1 and nursing field 2.

### 5.5 Comparison by Staff/Student and Gender

A Mann–Whitney *U* test was also applied to the data to determine whether any difference between the scores of academic staff and student respondents, and the scores of female and male respondents were of statistical significance regarding the perception of the suitability of men to the fields of nursing. While the test revealed that student respondents (Mdn = 7, IQR = 1, *n* = 51) perceived the field of adult nursing as less suitable for men compared to academic staff respondents (Mdn = 7, IQR = 0, *n* = 51, *p* = 0.01) the effect size was small (*r* = 0.28) (Cohen 1988) (Table 8). Results also show that student respondents (Mdn = 7, IQR = 1, *n* = 51) perceived the field of mental health nursing as less suitable for men compared to academic staff respondents (Mdn = 7, IQR = 0, *n* = 51, *p* = 0.02), and the effect size was small (*r* = 23). No statistically significant difference between the female and male respondents' perception of the suitability of men to the fields of nursing was observed (Table 9).

**TABLE 8:** Mann–Whitney *U* test results comparing the perception rating of male suitability to each field of nursing practice between the female and male respondents.

Nursing field	Respondent group	*N*	Mean rank	*u*	*z*	*p*	*r*
Adult	Student	51	45.29	984	−2.78	0.01	0.28
	Academic	51	57.71				
	Total	102					
Child	Student	50	46.36	1043	−1.65	0.10	0.17
	Academic	50	54.64				
	Total	100					
Learning disability	Student	50	46.46	1048	−1.72	0.08	0.17
	Academic	50	54.54				
	Total	100					
Mental health	Student	51	46.60	1050.5	−2.29	0.02	0.23
	Academic	51	56.40				
	Total	102					

*Note:* Results of testing the null hypothesis that there is no difference in the perception of male suitability to the various fields of nursing practice between the student and academic participant groups.

Abbreviations: *U* = primary test statistic, *z* = standardised test statistic, *r* = effect size according to Cohen (1988), the *p*‐value (*p*) significance level is set at equal to or less than 0.05.

**TABLE 9:** Mann–Whitney *U* test results comparing the perception rating of male suitability to each field of nursing between the academic nursing staff and nursing student respondents.

Nursing field	Respondent group	*N*	Mean rank	*u*	*z*	*p*	*z*
Adult	Female	63	51.40	1222	−0.06	0.95	0.01
	Male	39	51.67				
	Total	102					
Child	Female	61	53.48	1008	−1.47	0.14	0.15
	Male	39	45.85				
	Total	100					
Learning disability	Female	62	52.85	1032.5	−1.26	0.21	0.13
	Male	38	46.67				
	Total	100					
Mental health	Female	63	52.89	1141	−0.81	0.42	0.08
	Male	39	49.26				
	Total	102					

*Note:* Results of testing the null hypothesis that there is no difference in the perception of male suitability to the various fields of nursing practice between the female and male participant groups.

Abbreviations: *U* = primary test statistic, *z* = standardised test statistic, *r* = effect size according to Cohen (1988), the *p*‐value (*p*) significance level is set at equal to or less than 0.05.

### 5.6 The Perception of Accessibility to Specialised Areas of Nursing for Men

Table 10 shows the median scores for each specialised area of nursing. The pathways rated most accessible were “leadership and management,” “acute and emergency care,” “prison services,” “education and training,” and “research and development” (Md = 7). Next were “acute wards” “commissioning care,” and “intensive and critical care” (Md = 6). The pathways rated least accessible were “care of a newborn baby” (Md = 3), followed by “residential or longer‐term care,” “palliative/end of life care,” “community and primary care,” and “public health” (Md = 5).

**TABLE 10:** Respondents median scores rating male accessibility to specialised fields of nursing.

Specialised field of nursing practice	All respondents			Student respondents			Academic respondents			Female respondents			Male respondents		
	*N*	Mdn	IQR	*N*	Mdn	IQR	*N*	Mdn	IQR	*N*	Mdn	IQR	*N*	Mdn	IQR
Acute and emergency care	104	7.00	1	38	6	2	40	7	1	44	7	1	33	7	2
Acute wards	103	6.00	2	38	6	2	40	7	1	44	7	1	33	6	2
Care of newborn baby	100	3.00	3	38	3	2	40	3	2	44	4	3	33	3	2
Commissioning care	95	6.00	1	38	6	2	40	7	1	44	7	1	33	6	2
Community and Primary care	102	5.00	3	38	5	2	40	5.5	3	44	6	3	33	5	2
Education and training	104	7.00	2	38	6.5	3	40	7	1	44	7	1	33	6	3
Intensive and Critical Care	103	6.00	2	38	6	2	40	7	1	44	7	1	33	6	2
Leadership and management	106	7.00	1	38	6	2	40	7	1	44	7	1	33	6	2
Palliative/end of life care	100	5.00	3	38	5	3	40	6	3	44	6	3	33	5	3
Prison services	100	7.00	1	38	6.5	2	40	7	1	44	7	1	33	7	1
Public health	101	5.00	3	38	4	2	40	5	4	44	5	3	33	4	2
Research and development	102	7.00	2	38	6.5	2	40	7	1	44	7	1	33	6	2
Residential or longer‐term care	103	5.00	3	38	5	3	40	5.5	3	44	5	3	33	5	3

Abbreviations: IQR, nterquartile range; Mdn, median score; *N*, total number of respondents.

### 5.7 Content Analysis Findings

While some respondents held the view that there are no barriers to men at the point of entry to any field of nursing or progressing in a nursing career, for many, however, the proviso was that if there is a problem it was due to societal stereotyping and stigmatising, rather than a reflection on men in general:

I think that there may be some hesitation by employers when considering the care of children, which is a reflection on the societal concerns with risk, rather than on men's abilities to provide care. (I would prefer to see whether the respondent was student/staff and also the gender may be relevant when discussing perspectives). (Adult Nursing lecturer, female, respondent 1)

An overarching theme was found that represented perceptions of accessibility and suitability of men in nursing, “*stereotyping and stigma about men as nurses*,” which permeated the sub‐themes of; “*distrust of men*,” “*career progression in nursing is advantageous to men*,” and “*appeal of nursing as a career for men*.”

### 5.8 Stereotyping and Stigma About Men as Nurses

Multiple negative, and often discriminating beliefs, were reported about a societal perception of men as nurses, based on a belief that nursing is a feminised identity relating to caring and that men may have limited ability to care. This was particularly in relation to the field of child nursing where “*children nursing is associated more with motherhood*” (Adult Nursing lecturer, male, respondent 18), and that females have a high caring proficiency whereas men have a caring deficiency, and therefore “*caring environments are more difficult for men to gain acceptance or to be seen as competent in comparison to female colleagues*” (Mental Health lecturer, male, respondent 33). This feminised identity was also linked to female‐focused messages and the use of role descriptions, such as “*Sister for instance, with no term equivalent for men*” (Adult Nursing lecturer, male, respondent 36). There was also a perception that the media portrayal of men is unsympathetic:

I feel that some of the fields aren't as accessible as not all women feel safe or comfortable around men due to social and cultural challenges that are seen in media. (Undergraduate Adult Nursing student, male, respondent 70)

Furthermore, some reported that men may be seen to have stereotypical character features, or a number of hidden agendas in terms of pursuing a career in nursing:

…there is a fear/stigma that men wanting to forge a career in child nursing either have a negative ulterior motive, could be homosexual, have other underlying issues including mental health, lack of confidence, etc. (Undergraduate Mental Health Nursing student, male, respondent 52)

These perceptions were believed to have led to a distrust of men as nurses in some fields, although predominantly child nursing that may explain some of the statistical data revealing that child nursing is perceived to be the least accessible to men.

### 5.9 Distrust of Men as Nurses

Stigmatisation and stereotyping suggested a distrust of men working in the nursing workforce and that men were predisposed to inflict abuse:

[there is] the perception that some men are potential abusers or may be inappropriate with patients, whether they are adults or children. (Adult Nursing lecturer, male, respondent 4)

A sense of being distrusted impacted on the experiences of men during their education, particularly within childcare placements where they were instructed:

Don't pick a child up, don't toilet a child, don't cuddle a child, don't change a nappy without a chaperone etc. It scared me to death!! As a man, I feared being a children's nurse would make people suspect me of having the wrong intentions. (Adult Nursing lecturer, male respondent 22)

I have heard from fellow male children's nursing students that they don't feel as trusted with patients as female children's nursing students. (Undergraduate Learning Disabilities Nursing student, male, respondent 30)

It was also believed that men may, potentially, not be trusted due to previous traumatic experiences of patients, that had involved men as perpetrators:

it may be harder for children or adults with bad experiences or trauma from men to accept care. (Undergraduate Adult Nursing student, female, respondent 35)

The combination of distrust of men as nurses and associated stigma and stereotyping led to a belief, for many of the responders, that nursing may not be a suitable career for men. Nursing, as a profession unsuitable for men, was not only linked to stigma and distrust, it was also linked to experiences such as; “*I have found being male, some of the older female patients do not like male personal care or other cultures due to their religion*” (Undergraduate Adult Nursing student, male, respondent 49). Also, in delivering personal care in the environment of an individual's home many individuals would rather trust a female nurse then a male nurse, particularly where washing, dressing, and catheter care for older women was required, “*I have experienced a few occasions where ladies have asked for the males not to do it*” (Adult Nursing lecturer, female, respondent 20).

Paradoxically, although there was significant reporting that nursing may not be a suitable career for men, gendered stigma and stereotyping (in favour of men) also impacted on beliefs and perceptions that men progressed to more senior roles in nursing much easier than women.

### 5.10 Bullying and Male Nurses

Some respondents deemed men to be susceptible to incidents of bullying. This may come from female colleagues towards junior male nurses or from patients with a prejudicial view of men being nurses. Men were described as having “*to go extra mile to fit into the team*” (Undergraduate Child Nursing student, female, respondent 94). A stereotype that men are more emotionally resilient and can “*handle it better*” may well be a factor here:

Emotional abuse, bullying, discrimination, often come from female colleagues, especially if male nurses are at lower bands. (Adult nursing lecturer, male, respondent 36)

With bullying it's because they don't see male nurses very often. So they may make fun of them. They may also give them abuse due to the fact they think they can handle it better. (Undergraduate Child Nursing student, female, respondent 94)

### 5.11 Men Associated With Leadership and Technology

There was repeated reporting of the visibility of men in more senior roles as well as acute and critical care roles. These roles were less about hands on personal care nursing activities and more about acute and emergency environments with technology, strategy, decision‐making, and leading a service. Men were also described as occupying roles where career progression happens more quickly:

Leadership/management and roles that require technical competence are seen as more appropriate to gender ideals associated with men. (Mental Health Nursing lecturer, male, respondent 33)

Senior and more acute roles appear to be filled by men who progress quicker. Roles with less progression seem to be more female dominated. (Undergraduate Adult Nursing student, female, respondent 68)

There is a higher proportion of men in education, leadership and commissioning roles in nursing than in nursing overall. (Adult Nursing lecturer, female, respondent 24)

I tend to see male nurses disproportionately in acute and emergency care, and in leadership roles. (Adult Nursing lecturer [gender not disclosed], respondent 11)

In my personal experience I have seen a high incidence of men in critical care, leadership and Acute emergency medicine. (Adult Nursing lecturer, female, respondent 80)

From observation only. i.e., for prison services, A&E more men are present when I have completed shifts; also, for education, research and leadership roles [I have] observed more men in these areas. (Child Nursing lecturer, female, respondent 13)

Men were described as less visible in child, learning disability, neonatal care, care homes, palliative care, sexual health, and women's health‐related roles. Descriptions of male visibility or lack of in particular fields and pathways in the open text data aligned with fields and pathways considered accessible or less accessible to men in the quantitative data.

In keeping with being visible in senior roles, men were seen as having a career advantage that favoured them reaching senior positions which was assigned to: career breaks and childcare as being less impactful on the career progression of a man than a woman; working term times or part time to cover childcare; less need for consideration over issues such as maternity leave, childcare issues; a perception that men are driven to progress to higher salaries, “*men's pay is poor working as a nurse*” (Undergraduate Mental Health Nursing student, female, respondent 75).

Not only in leadership and management, but it was also reported that research career pathways favoured men over women; “*in some areas such as research, there is a path and it is loaded towards men*” (Mental Health Nursing lecturer, male, respondent 31). Furthermore, there was also a perception that “*once you have qualified, getting on is usually easier for men*” (Child Nursing lecturer, male, respondent 5). The male friendly clinical roles and careers, as opposed to management positions, that were viewed as favouring men were predominately in mental health services, prisons, and emergency care. “*From my experience all of the Mental Health Nurses I have met have been male. For Adult Nursing I think it depends on the specialty, ITU and ED had more male Nurses whereas there were fewer male Nurses on the wards*.” (Adult Nursing lecturer, male, respondent 57).

### 5.12 The Appeal of Nursing as a Career for Men

The fields of nursing and specialised areas where men were most visible were also deemed to be most appealing to men. The perceived appeal was gendered, in so much as there was a belief that some areas of practice were more “*exciting*” and “*macho*,” and environments where there was a lot technology and machines were seen to be areas attractive to males:

I have found more male nurses in certain roles than others—ED, ITU, research had more male nurses. Whereas district—community nursing had far less. I think it's down to perception—ITU and ED are exciting, fast‐paced areas with lots of clinical procedures, not knowing what's coming through the door makes it exciting. (Adult Nursing lecturer, male, respondent 57)

It was suggested that fast‐paced and exciting roles appealed to male character traits “*male nurses may be attracted to prison services due to the stereotype and expectations of men being ‘strong’ and ‘tough’*” (Undergraduate Adult Nursing student, male, respondent, 64), and that “*sedate*” and “*slower*” ward roles may be unappealing.

Mental health nursing was viewed as usually featuring men at the forefront, typically highlighting the “*perceived physical demands*” in relation to caring for challenging clients who may require physical and chemical “*restraint*” for instance (Adult Nursing lecturer, male, respondent 36). Stereotypically such actions would be the role of a male to undertake due to the potential “*violent*” nature of the action. It was believed that this could be appealing to men, in much the same way that prison nursing may be attractive to men.

However, further confirming stereotypical assumptions, some respondents revealed quite the opposite appeal of both prison and acute mental health environments for men:

I have a friend who is a gay male nurse and he got a job at a prison in [name of prison] and he was actually scared to go to work even though it was something he wanted because he was scared of how he would be treated and possibly threatened. (Undergraduate Adult Nursing student, female, respondent 25)

In accordance with the stereotyping of nursing was the implication that environments considered as female dominated can be unappealing to men, “*I think the proportion of women to men* [in nursing] *is off‐putting*.” (Undergraduate Mental Health Nursing student, male, respondent 82).

## 6 Discussion

While the under‐representation of men working in nursing in the United Kingdom is long‐standing and well documented (Nursing and Midwifery Council 2022; Universities and Colleges Admissions Service and Health Education England 2021; Whitford et al. 2020; Clifton, Crooks, and Higman 2020), this study provides insight into the perceptions of accessibility and suitability of men to the specific nursing fields and career paths, and the perceived barriers that exist to men entering the nursing profession from the perspective of both staff and students in Schools of Nursing embedded within HEIs in England.

### 6.1 Fields of Nursing Practice, Specialised Areas, and Male Accessibility

Overall, results from the quantitative element of this study indicated that the fields of adult and mental health nursing are considered the most accessible to men entering the profession and child nursing considered the least. The quantitative results also revealed particular nursing career pathways to be perceived as more accessible to men: leadership and management, acute and emergency care, prison services, education, research roles, acute wards, commissioning care, and intensive and critical care were all rated by respondents as more accessible to men than roles in neonatal, residential and palliative care, community and primary care, and public health. Supporting this study's findings, a longitudinal study by Mclaughlin, Muldoon, and Moutray (2010) reported that nursing students in the United Kingdom considered particular nursing roles as being more accessible to men including mental health, accident and emergency, learning disability, theatre, surgical, medical nursing, management, and teaching roles. Conversely, Mclaughlin, Muldoon, and Moutray (2010) found nursing roles in school, district, health visiting, child, and practice nursing to be considered as less accessible to men and deemed “*highly feminine*.”

The question remains why some nursing roles are perceived as more accessible to men than others. Nursing is a complex and dynamic profession characterised by a range of roles and expertise. As with other gender‐specific occupations such as Science, Technology, Engineering and Math (STEM) fields (Robnett 2016), the social perception of roles will impact the gender make‐up of the student nursing population and consequently the nursing workforce. Carryer (2022) argues that gender, as a social construct, is central to how nursing is viewed socially with gender‐specific roles extending into nursing as a profession. Elsewhere the societal view of nursing as a gender‐specific profession is said to be outdated, poorly informed and not an accurate reflection of the modern‐day profession that nursing has become (Garcia and Qureshi 2022). A study with nursing students in Australia found students, regardless of gender, largely reject misconceptions of men working as nurse, and the idea that men lack the appropriate qualities required to work as a nurse (Ramjan et al. 2024), although students that did hold stereotypical views of men working as nurses were more likely to be held by male nursing students.

Our study highlighted the view that environments in nursing where technology is prominent, such as in ITU and ED, are appealing to men based with such areas described as being “*technical*,” “*exciting*,” and “*fast‐paced,”* a view that is supported elsewhere in the literature (Whitford et al. 2020; Guy, Hughes, and Ferris‐Day 2022; Evans 1997; DeVito 2016). Indeed, Guy, Hughes, and Ferris‐Day (2022) reported that recently qualified men working as nurses felt the profile of nursing pathways characterised as technical, including emergency, critical, and perioperative care, needed to be raised as a means of attracting more men to the profession. In terms of their future selves, male nursing students have been shown to see themselves occupying roles of a leadership or technical nature (Prosen 2022). Elsewhere findings revealed that male nursing students engaging with the technical elements of nursing were associated with higher retention rates for undergraduate male nursing students (Stott 2007). Contrary findings have however suggested that progressing vertically into a management role was not found to be appealing for male nursing graduates who undertook nursing as a second degree; these graduates were more focused on being personally fulfilled by their career, by providing a direct nursing care to help and support their patients (Harding et al. 2018).

### 6.2 Stereotyping and Stigma

Our qualitative results found stereotyping and stigmatising at a societal level creates barriers to men entering the nursing profession and is influential in terms of the roles men take up after entering the profession (Harding, North, and Perkins 2008; Brody et al. 2017). Our results convey multiple negative and discriminating stereotypes about men working as nurses relating to the societal perception that nursing is a gender‐specific profession. The long held stereotypical view persists of men who are nurses as effeminate or homosexual, although traditionally men were not given space within traditional nursing imagery, which tended to focus on the perception of nurses being “*angels*” or “*sex objects*” (Teresa‐Morales et al. 2022).

Roles in prison services and mental health services were described using stereotypical assumptions related to men being “*strong*” and “*tough*,” and being able to deal with some of the more physically challenging, and possibly violent, aspects of the environments. However, respondents in this study expressed gay men feeling at risk of violence in both prison and acute mental health environments because of their sexuality, supporting the view that discussions relating to gender are complex and layered (Lorber 2018).

“*Feminine*” traits, such as sympathy and understanding have been described as fundamental to delivering care and that attracting men willing to display such traits might not just attenuate recruitment and retention issues affecting men working as nurses, but also contribute to addressing the issues of stigma surrounding men occupying careers in care more broadly (Teresa‐Morales et al. 2022; Loughrey 2008). On the other hand, certain pathways; including mental health, accident and emergency, learning disability, theatre, surgical, medical nursing, management, and teaching roles (Mclaughlin, Muldoon, and Moutray 2010), and areas associated with the technical aspects, that is, where technology is prevalent, are described as being more appealing to men (DeVito 2016; Stott 2007) and these could be used to create a more inclusive environment for male students (Whitford et al. 2020; DeVito 2016). Nursing therefore need not be portrayed to the potential future workforce homogenously in terms of the skill set and working environment, as being solely either “feminine” or “masculine.”

A societal approach to and perception of risk has led to a distrust of men and their intentions when entering a nursing career, particularly with regard to vulnerable patients (Clifton, Crooks, and Higman 2020). Our data showed the distrust of men to be specifically associated with career potential in the field of childcare, where stigmatisation and stereotyping suggested that men were predisposed to inflict abuse. There were also reported experiences by students within placements with children, where instructions were given that created anxiety and fear about any contact they may have with children and babies, perpetuated by senior staff within those clinical environments. This study also found stigma and distrust were linked to carrying out intimate care for females, in any setting, for both potential embarrassment‐related reasons, and also for female patients from cultures where their religion precluded having men undertaking personal care. Consequentially, respondents perceived the different nursing fields of practice as accessible or suitable to men due to being limited in the tasks they can carry out and the nursing fields of practice they can work in. Analysis by Harding, North, and Perkins (2008) reported that culturally, while physical touch by women in nursing is interpreted as a caring act, for men in nursing it is problematic and sexualised. That said, the General Medical Council (2024) have produced guidance for all healthcare professionals which covers and considers some the issues prior to any sensitive examinations for patients by clinicians of all genders.

Experiences reported in our open‐text data indicated a level of lateral violence occurring against men working as nurses. While bullying is not exclusive to men working as nurses and occurs at all organisational levels (Wilson 2016), higher rates of bullying have been reported by male staff and staff with a disability than other demographic groups within the NHS workforce (Carter et al. 2013). Bullying has been found occurring for staff transitioning from student nursing to registered nursing (Randle 2003), and Bouret and Liners Brett (2017) found some unique lateral violent behaviours towards men being experienced in both nursing educational and practice settings for men working as nurses in Canada. This included exploitation of physical strength, repeatedly harassed for being “effeminate,” and non‐verbal innuendos. Our findings do indicate a need for further study on men's experiences, specific to the field of children's nursing and potential features of lateral violence in this field.

In a historical review of men in nursing in Canada, the United Kingdom, and the United States, Evans (2004) points out that both social and political factors, and prevailing notions of masculinity and femininity, have shaped men's position within the nursing profession. She posits that “*the high status of men relative to women has had significant implications for the career paths of men*,” and offers “*insights into the ways in which gender/power relations have excluded, limited or, conversely, advanced the careers of men nurses*” (p. 327). Data in this study did suggest that, for some nursing roles, stereotyping favours men, in that there was a strong belief and perception that career progression in nursing is advantageous to men and that men progressed to more senior roles in nursing much easier and faster than women. This perception is also supported by Punshon et al. (2019) who reported that in the NHS men have been found to be advantaged in terms of pay; are over‐represented at senior Bands, compared to their overall percentage of nurses in the United Kingdom; and also appear to be advantaged in that, from the point of registration, they progress to higher staff positions faster than their female colleagues. This has been termed the “*glass escalator*” (Williams 2013) in which the structural advantages men hold in wider society are replicated within the nursing profession to the detriment of women within the nursing workforce (Clifton, Crooks, and Higman 2020). An integrative review by Smith et al. (2021) found that men working as nurses find the sector to be a “double‐edged sword” where the result of being a visible minority brings about both challenges and successes; the review found that visibility can be advantageous, leading to career progression while also subjecting men to increased prejudice and scrutiny.

Respondents in this study perceived that the reasons men progressed to management and leadership roles in greater proportions and with greater ease than their female counterparts were principally due to career breaks and challenges such as maternity leave and childcare being less impactful on the careers of men compared with the careers of women. A global systematic review, which included studies from the United Kingdom, on women and nursing leadership (Baduge Pincha et al. 2023) identified a number of barriers to women including expectations from society, organisation processes and policies, gender‐bias, lack of access to training, and a lack of family friendly working arrangements hindering access to development opportunities. Baduge Pincha et al. (2023) found that the careers of women progressed quickly when they were well supported and received training and development when returning from parental leave. Conversely, in the United Kingdom, women were most likely to leave the acute nursing field in the NHS when aged 30–34, with caring responsibilities cited as a likely reason, compared with men who left this field in greater numbers aged 55 and above (Kelly, Stoye, and Warner 2022).

## 7 Conclusion

This study aimed to examine the accessibility of men into nursing profession as perceived by those currently studying or teaching nursing in England. The nuanced findings revealed that gender bias is perceived to be persisting in areas of practice that are considered to be more accessible for women who are nurses, for example, child nursing. Concomitantly, other fields of practice, for example, mental health nursing, are associated with masculine traits therefore more suitable for men working as nurses. While nursing as a profession is complex with characterised pathways, roles and expertise that are diverse, our results found persisting stereotypes about men. Men are perceived as a risk and there is a distrust of their intentions, particularly relating to caring for vulnerable members of society such as children. The negative and discriminating social perceptions of men relating to nursing roles will impact the gender make‐up of the student nursing population and consequently the nursing workforce. This study potentially informs the development of recruitment strategies in nursing that tackle these discriminatory views, alongside educational initiatives for promoting nursing as a career for men.

This study provides examples of how stereotyping, stigma, and social perspectives relating to gender may impact the pursuit of a career in nursing for men. The evidence produced can be used to inform nursing recruitment initiatives that are more inclusive in nature, particularly in regards to presenting a more accurate and appealing picture of the diversity of environments, roles and career paths available within the nursing profession. When recruiting into the nursing sector better highlighting of skills that are described as having more appeal for men, such as the more technical specialities, could be a means to creating a more inclusive portrayal of the sector. Ultimately, men working within the nursing workforce can contribute to the sector both quantitatively by addressing current staff shortages within the sector, and also qualitatively through a diverse skillset.

### 7.1 Limitations and Strengths of the Work

Respondents were geographically diverse, coming from a variety of HEIs located across England, providing views from both staff and students from these institutions. While all Nursing schools in HEIs based in England were invited to respond to the questionnaire, not all were represented, as circulation to the staff and students at each school was at the discretion of the Head of the respective school, to whom the study invitation was sent. As such, a large number of students and staff studying and teaching nursing in England did not participate in this study. The sample is self‐selected which maximised the opportunity for members of the target population to engage with the study. However, there is the potential that bias was a motivation factor for those choosing to respond in that those who chose to respond already held strong views on the topic of study. In terms of the methodology, the open‐ended question data collected was detailed and subjected to a rigorous content analysis process. The questionnaire aimed to capture perceptions of men's accessibility to the nursing profession. However, the questionnaire pilot was limited and it is therefore recommended that there is further research and development of a questionnaire designed to measure accessibility to the nursing profession, with an expanded pilot to better measure construct validity.

### 7.2 Recommendations

As a means of tackling the long‐standing stereotype that the nursing profession is not for men, universities must design recruitment strategies aimed at future nursing students that better promote the diversity of career pathways within the nursing sector. The sector is defined by its provision of person‐centred care which is delivered in many ways and diversifying the portrayal care delivery may better reflect the diversity of the potential experiences available to those choosing a nursing career. For example, increasing the promotion of the “*technical*,” “*exciting*,” and “*fast‐paced*” aspects of care in environments such as those in ITU, ED, and perioperative care, may widen the appeal of a nursing career to more men. Also, increasing the promotion of men working in child nursing to the potential future workforce may help to tackle stigma in this field. Publishing the evaluation of recruitment strategies would be beneficial. Future strategies must also be directed at removing barriers women may face accessing certain nursing career pathways such as those in leadership or research. Further support is recommended for male students in order to combat feelings of isolation, bullying, and lower completion rates for men on nursing programmes. Creating, promoting, and evaluating the value of support groups for male students in both academic and clinical settings is recommended as a forum for male students to share experiences. Furthermore, research into the experiences of male paediatric nursing students and nurses would be beneficial.

## Author Contributions

Made substantial contributions to conception and design, acquisition of data, or analysis and interpretation of data. D.C., L.H.M., and K.D.V. involved in drafting the manuscript or revising it critically for important intellectual content. D.C., L.H.M., K.D.V., A.C., G.M., and J.S. given the final approval of the version to be published. Each author should have participated sufficiently in the work to take public responsibility for appropriate portions of the content. D.C., L.H.M., K.D.V., A.C., G.M., and J.S. agreed to be accountable for all aspects of the work in ensuring that questions related to the accuracy or integrity of any part of the work are appropriately investigated and resolved.

## Conflicts of Interest

The authors declare no conflicts of interest.

## Peer Review

The peer review history for this article is available at https://www.webofscience.com/api/gateway/wos/peer‐review/10.1111/jan.16789.

## Statistics

The statistics presented in this article have been checked by the following author George McGill who is a statistician.

## Data Availability

The data that support the findings of this study are available from the corresponding author upon reasonable request.
